# Commentary on a participatory inquiry paradigm used to assess EOL simulation participant outcomes and design

**DOI:** 10.1186/s13584-017-0187-7

**Published:** 2017-11-20

**Authors:** Jane M. Gannon

**Affiliations:** 1 University of Florida Health Science Center, Jacksonville, USA; 2 College of Nursing, Learning Resource Center, 653-1 West 8th Street, Box L-4, Jacksonville, FL 32209 USA

**Keywords:** Simulation, End-of-life care, Simulation design, Simulation evaluation, Health sciences

## Abstract

Care at the end-of-life has attracted global attention, as health care workers struggle with balancing cure based care with end-of-life care, and knowing when to transition from the former to the latter. Simulation is gaining in popularity as an education strategy to facilitate health care provider decision-making by improving communication skills with patients and family members. This commentary focuses on the authors’ simulation evaluation process. When data were assessed using a participatory inquiry paradigm, the evaluation revealed far more than a formative or summative evaluation of participant knowledge and skills in this area of care. Consequently, this assessment strategy has ramifications for best practices for simulation design and evaluation.

## Commentary on a participatory inquiry paradigm to assess participant outcomes and simulation design

The transition from acute, cure based care to end-of-life (EOL) comfort based care represents a challenge to all health care providers (HCPs). While technological advances provide a multitude of life extending treatments, their use often conflicts with what the patient wants as their life force wanes. Further, health care professionals must often struggle to overcome their own value system and even the Hippocratic oath for some, when recognizing that a transition from cure to care is warranted. Family members also play a role in either supporting or hindering the decision-making process during this care transition, and communication is essential in developing a mutually agreeable care management plan.

The need for purposeful, well-timed, and compassionate EOL care is a global issue that many are working to improve. This is especially true in the transition from curative to comfort-based care. Despite proliferation of end-of-life care options, the dying process in the U.S. is expensive, focuses on aggressive efforts to extend life, and lacks coordinated care delivery. The major reason for this is a lack of preparedness of health care providers being able to effectively communicate and collaborate during this care transition [1].

In the U.S., there are multiple organizations and systems calling for better training and preparedness of health professionals to manage EOL care. These include the American Association of Colleges of Nursing [2], the Strategic Planning Summit for Pain and Palliative Care Pharmacy Practice [3], and the Liaison Committee on Medical Education [4]. In our setting at the University of Florida-Jacksonville campus, representatives of each of these professions (nursing, pharmacy and medicine) collaborated on an interprofessional simulation activity that compared the simulation approach to a paper case-study approach. We found the experiential learning process offered by simulation to be superior to the case study method in changing attitudes toward EOL care [5].

In their IJHPR article, “Simulation Based Training for End-of-Life Care,” authors Brezis and associates describe a national initiative in Israel undertaken to improve communication skills in HCPs involved in EOL care using simulation as the education strategy [6]. A mixed methods approach was undertaken to examine phenomena in the simulation experience that impacted how HCPs interact with clients at this precarious and often crisis-ridden time. Their study, which started off as an educational initiative to improve HCP communication at EOL, transformed into a qualitative inquiry about the behaviors and communication patterns that HCPs demonstrate in uncomfortable situations like EOL care discussions.

The authors describe the process used by a steering committee at the Israel Center for Medical Simulation to develop a series of six scenarios that formed the basis for an EOL care workshop. Health care teams from across Israel were invited to participate in the educational offering. Initially planning to focus on measuring the impact of the simulation experience on satisfaction, attitudes and other perceptions using typical research survey tools, investigators found themselves awash in data from which they realized other investigative strategies were needed for interpretation. Qualitative approaches, including deliberative dialogue strategies to invoke reflection and reframing of the simulation experience were used in combination with the questionnaire data to achieve understanding of the phenomenon, until “theoretical saturation” was accomplished.

This study offers insights into not only the rich education experience in EOL care that simulation provides for the participant through understanding, performing and caring, but also what it offers the educator/ facilitator. The authors realized the need to go beyond the use of validated tools for outcome measurement, and so eventually incorporated the use of a participatory inquiry paradigm, to gain insight for themselves into how context was interpreted by participants, and how they made connections between content areas.

The case for a participatory inquiry paradigm to guide the evaluation of their simulation effort stems from work by John Heron and Peter Reason [7]. Heron and Reason emphasized the important role experiential learning plays in understanding the world around us. The use of interprofessional groups engaging in simulation reflect Heron and Reason’s collaborative forms of inquiry. Integrated into the participatory inquiry paradigm are Guba and Lincoln’s three approaches (ontological, epistemological and methodological) that guide understanding of reality encountered during the learning process [8].

In this study, not only is the experience of the students critical to understanding the nature of the end-of-life experience for the patient, but so too is the student experience important to faculty evaluators in understanding the student learning experience. During the simulations themselves, students attempt to answer the ontological question, “What is the form and nature of reality and, therefore, what is there that can be known about it?.” [8] However, those faculty members charged with evaluating student performance become part of that paradigm as they seek to answer the epistemological question, “What is the relationship between the knower or would-be knower and what can be known?” [7] Faculty evaluators, as “knowers” in their own right, are in a unique position of being able to observe would-be knowers as students experience the simulation. This provides evaluators a unique perspective of the student learning process.

As such, this study has much to offer not only to those engaged in EOL based simulation, but in fact, any educational strategy in which health care-based events are simulated. Such strategies provide the opportunity for assessing, observing and/or understanding the learning process that simulation offers, its impact on participants, and how design changes can be made to facilitate participant performance.

Because of their approach to assessment, the study has ramifications for not only how best to assess those exposed to simulations but also how to improve a simulation’s design. The International Nursing Association for Clinical Simulation and Learning (INACSL) Standards of Best Practice describe the criteria for ensuring that simulations are designed to meet identified objectives [9]. From the evaluation perspective of the simulation activity itself, this includes evaluating the simulation-based experiences to facilitate design changes. While criterion #9 in the Standard stipulates that assessment data be used as part of a quality improvement approach to program evaluation, specific guidance is lacking [9]. Brezis’ study however, offers one such avenue that can be pursued as an evaluative approach to both simulation design as well as participant evaluation.

Typical simulation-focused evaluation tools include check-lists, attitude perceptions, knowledge, and behavior based changes. Higher level evaluation even measures the impact the learner exposed to simulation makes on patient oriented outcomes. While significant attention in recent years has focused on the use of debriefing to promote reflection, structured approaches are advocated, like Delta Plus [10], PEARL [10], and Advocacy/Inquiry method [11]. Such structured approaches may be limiting in what can be revealed about simulation design features that impact participant decision-making and problem-solving processes.

In this study, the authors suggest other important avenues of assessment to pursue, either in the education or research domains. Using a participatory inquiry paradigm combined with qualitative thematic analysis, the team sought to extract insight from participants. Examining questionnaire-based data and combining it with what can best be described as a group reflection process, the authors were able to learn more about why the participants performed as they did. That includes understanding why they did not use evidence-based opioid administration guidelines, or why they had a lack of awareness of legal and ethical principles related to EOL care, and lacked understanding of the dying process, among others.

The team went on to make use of video recorded sessions for an RIAS-based thematic analysis of communication components. Typically, such videos are utilized in the debriefing process, to describe participant actions for example, or to differentiate what went well from what could be done differently. As a source of assessment data, the team used them to examine the communication patterns of HCPs, specifically looking for cognitive and affective utterances. The differences in communication patterns between types of HCPs that were found in their analysis again provides valuable evidence for education needs in health science curricula across professions. There were particularly important learning needs found in listening and empathy skills. This helps inform not only health science educators of curricula needs, but also simulation design teams about a need for scenarios that can both facilitate and assess the performance of these skills. Our team on the University of Florida Jacksonville campus is using a similar approach to look for “huddle” based behaviors in a series of medication safety based scenarios. Additional analysis will examine the videos for team-based behaviors as these reflect the purpose of our simulation efforts.

The value of this study lies in showing educators how simulation can identify a wealth of learning needs, if that information is specifically sought. The study also provides evidence supporting the contention that the reflective process used during debriefing is at least as important as the simulation itself, if not more so. The simulation length in these scenarios were brief, around 7 min. The debriefing process lasted far longer.

According to INACSL’s debriefing standard, a required debriefing element calls for the use of a theoretically based debriefing framework and seven such frameworks are listed in the Standard [9]. Brezis and associates chose instead to use a participant inquiry paradigm approach. While clarity of the approach is lacking in the article, they do meet INACSL criteria spelled out in the Standard, including identifying contextual factors and clarifying the participant’s cognitive perspective that led to communication and other performance deficits. If the authors could better capture the structure to their approach, it would be a valuable addition to INACSL’s list of debriefing approaches.

## Conclusions

As noted by the authors, this participatory learning paradigm is reflective of an approach described in the literature as evaluation capacity building. Such an approach incorporates participant questioning to gather data that can be used for decision-making and action, and has greater value than the typical evaluation approach used in the simulation lab. It is not enough to simply run the simulation, check off a performance, or even provide a debriefing period. The reflection process utilized by this investigative team, combined with questionnaire data, provided insights into EOL education needs of health care providers across the State of Israel and a subsequent need to redesign both curricula and simulations to meet that need.

While much was learned about simulation experience and its structure, one must not forget that the focus of this initiative was EOL care. What started as a series of simulated scenarios in an EOL care workshop expanded to a national initiative that revealed a need for EOL culture change. As such, the assessment of data from the simulation events evolved into an ecological examination of how a cohesive structure for EOL care is lacking on the local, community and national level. This paper mirrors findings elsewhere, notably in the U.S where EOL care has gained new focus as HCPs grapple with the same issues as the authors.

## Abbreviations

EOL: End-of-life
HCP: Health care provider
INACSL: The international nursing association for clinical simulation and learning
PEARLS: Promoting excellence and reflective learning in simulation
RIAS: Roter interaction analysis system
